# Senemorphism: a novel perspective on aging patterns and its implication for diet-related biology

**DOI:** 10.1007/s10522-012-9383-6

**Published:** 2012-05-04

**Authors:** Lucas Siqueira Trindade, Alex Balduino, Toshiro Aigaki, Jonathan G. Heddle

**Affiliations:** 1 Heddle Initiative Research Unit, Advanced Science Institute, 2-1 Hirosawa, Wako, Saitama 351-0198 Japan; 2 Department of Investigative Pathology, Graduate School of Biomedical Sciences, Nagasaki University, Nagasaki, 852-8523 Japan; 3 Department of Biological Sciences, Tokyo Metropolitan University, 1-1 Hachioji, Tokyo, 192-0397 Japan; 4 Development & Technology Research Center, Universidade Veiga de Almeida, Rio de Janeiro, RJ 20271-020 Brazil

**Keywords:** Senemorphism, Caloric restriction, Longevity, Biomarkers, Senescence, Evolution

## Abstract

Aging can be described as the accumulation of changes in organisms over time. Aging in organisms undergoing caloric restriction (CR) is widely considered as a slowed version of aging under ad libitum (AL) conditions. However, here we argue that aging under optimized CR is fundamentally different from aging under AL based on the following facts: (1) Comparing the two dietary groups, several age-related changes run in the opposite direction over time; (2) Switching from an AL to a CR diet clearly reverts (not only delays) several “normal” accumulated changes; (3) major causes of death are as different between both groups as they are between species. These observations support the idea that CR and AL initially modulate different metabolic and physiological programs, which exclusively over time generate two biologically different organisms. Such distinct diet-related senescence is analogous to the divergent aging processes and causes of death observed between castes of social insects, such as queens versus workers (“caste-related-senescence”) and also between breeding versus non-breeding semelparous animals (“reproduction-related-senescence”). All these aging phenotypes are different not because they accumulate changes at a different rate, but because they accumulate different changes over time. Thus, the environment does not simply affect the individual aging rate through stochastic effects (e.g. U.V.) but also modulates the activation of a particular program/strategy that influences lifespan (e.g. caste, calorie intake). We refer to the environment-dependent aging patterns encoded by the genome as “senemorphism”. Based on this idea we propose experimental schemes for aging, evolution and biomedical research.

## Senemorphism (genetically molded aging patterns)

While aging appears to be an almost universal feature of life, aging rates and lifespans show great diversity even within a given species. A hypothetical example of a straightforward delay of an aging process would be where the process is the result of simple chemical reactions: A temperature-dependent difference in lifespan within species, for example, can be explained in part by the chemistry of life: lower temperature decelerates chemical reactions, resulting in an extended lifespan due to the slower speed of the degenerative processes (slower aging rate). In this case, the changes responsible for aging are supposed to be the same in the long-lived and short-lived individuals, only the reaction *rates* change. In other cases there appear to be two or more independent aging patterns, each dependent on differences in gene expression promoted by extrinsic factors. Here we demonstrate that most, if not all, species have such an adaptive and parallel evolution of age-related phenotypes. We propose the new term “senemorphism” to refer to a plastic aging phenotype which can be generated through genetic pathways in response to environmental conditions. This is a novel and vital concept, and the objective of this study is solely to: (a) highlight convincing evidence that longevity plasticity encoded by the genotype as a general phenomenon exists, and (b) to make the case that diet-related changes in aging are an important example of the same phenomenon. Based on this idea we will also allude to experimental schemes for aging, evolution and biomedical research.

In the following two sections we show that senemorphism exists by considering well-accepted examples of how aging is affected (via genetic pathways) by the environment. Note that, in this study, we will not attempt to propose an explanation for how or why different patterns of aging evolved.

## The case for senemorphism: caste

**Fig. 1:**
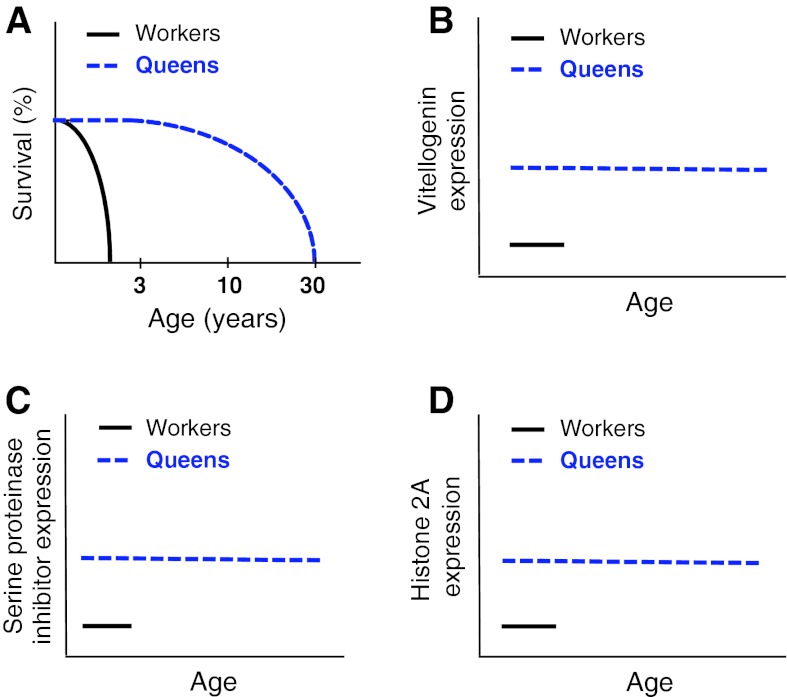
Differential gene expression controls caste-related senemorphism in ants (Gräff et al. 2007). **a**–**d** Lifespans and the constitutive higher gene expression related to longevity in ant queens (*blue*) compared to workers (*black*). **a** The longevity of *L. niger* workers versus queens (Gräff et al. 2007). **b** Different gene expression levels of vitellogenin over the lifespans of queens and workers. Increased vitellogenin expression is thought to lead to improved body maintenance and thus contribute towards increased lifespans. (Seehuus et al. 2006). **c** Relative expression of serine proteinase inhibitor genes in workers and queens. Higher levels of serine proteinase inhibitor are thought to be immunoprotective (Kanost 1999). **d** Histone 2A gene expression in queens versus workers. Higher histone H2A levels are thought to be responsible for improved maintenance of chromatin structure (Gräff et al. 2007). In addition, all of these genes are thought to have roles in strengthening the immune system

Senemorphism can be seen in social species, which are easily recognized by their adaptive and parallel evolution of age-related phenotypes. Different castes (soldiers, supersoldiers, workers, queens) evolved different lifespans, which most likely are correlated with their specific functions; as proposed by Hamilton (1966). *Lasius niger* sterile worker ants, for example, typically exhibit lifespans of 1–3 years whereas highly active reproducing queens with the same genetic background may live over 20 years (Keller and Genoud 1997). We refer to this phenomenon as “caste-related senemorphism” (Fig. 1).

## The case for senemorphism: reproduction

**Fig. 2:**
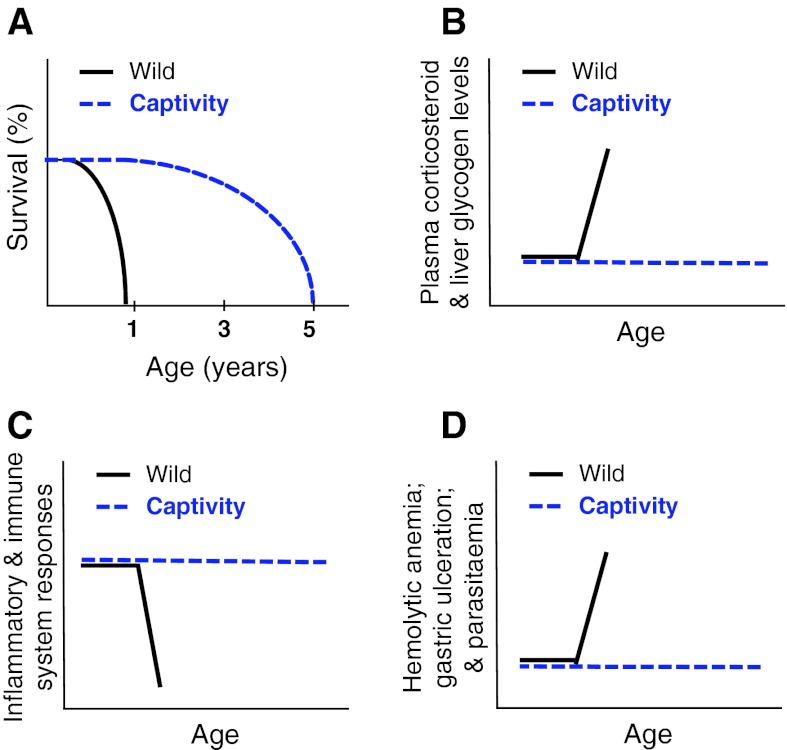
Evidence of two different genetic programs controlled by the environment generating different senemorphs. Life history death strategy during or shortly after a single breeding season is the lifespan-limiting factor for wild (*black*) semelparous animals such as semelparous dasyurids (Schmidt et al. 2006). In fact, semelparous animals have the potential to exhibit a normal aging process in captivity (*blue*). **a** Wild semelparous dasyurids live <1 year, dying mainly during the breeding season by activation of a death program. Captured animals are able to live up to 5 years (Bradley 2003). **b** Abrupt endocrine and physiological changes can be observed during death program activation, for example increases of cortisol and liver glycogen (Barnett 1973). **c** In wild animals, abrupt physiological changes cause diverse pathologies for example shut down of immune system and inflammatory responses (Barker et al. 1978; Cheal et al. 1976). **d** Remarkable increases of gastric ulceration, haemolytic anaemia and parasitemia observed in wild animals prior to death (Barker et al. 1978; Bradley 1997)

Reproduction-related senemorphism is most clearly seen in semelparous animals. Most semelparous mammals die during their first year, around 2 weeks after reproduction following an extremely rapid degenerative process. However, when reproduction is denied, the lifespan of these animals can increase several-fold and resembles a “normal” degenerative process (Schmidt et al. 2006; Woolley 1966). This we refer to as “reproduction-related senemorphism” (Fig. 2a). Functional disturbance of or removal of sexual organs in semelparous animals (as well as removal of the seeds from semelparous, annual plants) prolongs their lives (Robertson 1961; Wareing and Seth 1967; Wodinsky 1977). Interestingly, the females of some semelparous species of octopus die within days of hatching of their young, but removal of the optic glands after reproduction has taken place results in the reestablishment of normal behavior and increased lifespan (Wodinsky 1977). These examples show that the trigger that kills a semelparous animal is controlled at least partially by environmental conditions with the animals themselves in fact retaining the ability to maintain their physical integrity for longer periods. In other words, these animals can die due to a clear “death strategy” or die exhibiting a slower aging process. The death strategy of semelparous animals is related to extreme hormonal changes, increase of inflammation and a downgrading of the immune system (Humphries and Stevens 2001; Oakwood et al. 2001; Stein-Behrens and Sapolsky 1992; Wingfield and Sapolsky 2003). Non-breeding semelparous animals do not show these extreme changes because they undergo a different senemorphosis (Fig. 2b–d). In summary, there are several well-known species that show clear plasticity of lifespan due to changes in gene expression and hormonal levels, which appear to have evolved as an adaptation to a specific environment and result in at least two distinct routes to death.

## Diet-related senemorphism

In this section we present the case that diet (energy intake) is an important environmental factor determining aging phenotypes as is illustrated by the extreme differences between caloric restriction (CR) and ad libitum (AL) organisms.

**Fig. 3:**
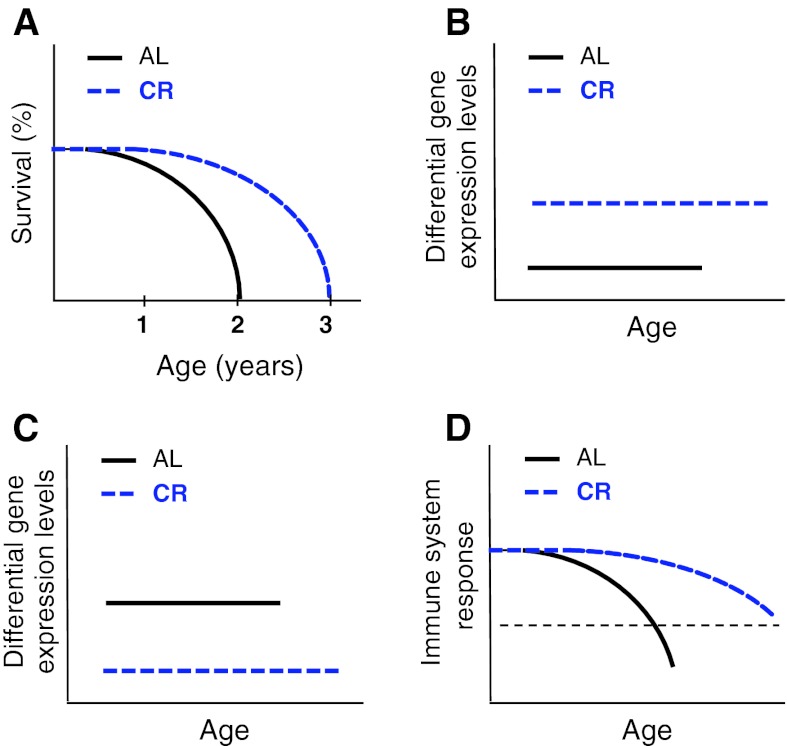
Comparison of lifespans and gene expression throughout total lifespan in AL (*black*) and CR (*blue*) mice, from birth to death based on the work of Cao et al. (2001). **a** CR mice live 50 % longer than AL mice. **b** Some genes (e.g. *Ubp1*, *Got1*, *Hal*, *Sultn*) remain expressed at a higher levels throughout life in CR mice. **c** Some genes (e.g. *hsp86*-*1*, *hspa5*, *ly6e*, *xbp1*) remain expressed at a higher level throughout life in AL mice. **d** Immune senescence is delayed by CR. Importantly, at the time of death the immune system most likely remains more efficient in CR animals (*dotted line*) (Chen et al. 1998)

The discovery that animals undergoing CR live longer than those undergoing AL (McCay and Crowell 1934) (Fig. 3a) was one of the most remarkable in aging research. The pathways involved in aging during CR or AL exhibit plasticity in response to extrinsic factors (Anderson and Weindruch 2007). They are ultimately controlled and coordinated by energy sensing mechanisms that are evolutionarily conserved from unicellular life forms to mammals (Flatt and Schmidt 2009; Fontana et al. 2010; Rose 2009; Rose and Nusbaum 1994). A commonly accepted hypothesis is that this response evolved to enable organisms enduring famine to survive until food returns and they can successfully reproduce (Holliday 1989). In contrast, under AL conditions reproduction is optimized at the expense of longevity (Kirkwood and Rose 1991; Holliday 1989; Masoro 2003; Merry and Holehan 1979). Because, generally, CR delays senescence compared with AL, the conventional conclusion is that the aging under CR conditions is simply a delay of the aging under AL conditions (as may be the case for a slower aging rate under low temperature). The alternative is that, as in social and semelparous species, aging under AL and CR are distinct processes related to metabolic differences rather than metabolic rate (Helfand et al. 2008; Braeckman et al. 2006). This hypothesis has been addressed experimentally in the case of short-lived animals (*Drosophila*). Mair et al. (2003) elegantly demonstrated that temperature and diet-related aging processes are fundamentally distinct: Whereas low temperature is related to a simple slowing in the mortality rate, calorie intake is clearly a diet-dependent adaptation of a specific lifespan strategy. Diet is already well known to influence the morphogenesis of organisms, affecting for example the final adult size, color, caste and even sexual differentiation. Here we highlight the commonly overlooked fact that the influence of diet extends to lifespan and remains throughout maturation, possibly until death. We call this “diet-related senemorphism.”

Diet-related senemorphism can be clearly identified through analysis of experimental data which shows clear and important differences between CR aging and AL aging. As in the case of the longer lifespan examples of caste and reproduction-related senemorphisms, CR animals show similar differences in gene expression, hormone levels and phenotypes (Cao et al. 2001; Higami et al. 2004) (Figs. 1, 2, 3). Several of these differences remain throughout the whole life of an organism or run in an opposite direction in CR compared to AL. CR animals can exhibit age-related changes and phenotypes that cannot be seen in AL animals even if diets are subsequently switched (Cao et al. 2001). These distinct diet-related aging processes are incompatible with the assertion that aging under CR is simply a delayed version of AL aging. Distinct aging processes are characterized by accumulation of distinct changes over time. By definition AL and CR aging are distinct processes and therefore examples of senemorphism. Evidence demonstrating diet-related senemorphism is discussed below.

## Evidence for diet-related senemorphism

In this section we present evidence to support the idea that CR aging is a fundamentally different process than AL aging and that different caloric intakes generate different senemorphs (specific aging phenotypes).

Anderson and Weindruch (2007) elegantly described metabolic reprogramming in CR, corroborating the idea that starvation generates different phenotypes. The fact that CR and AL phenotypes are completely different does not exclude the possibility that the age-related changes could occur in the same direction, but at a slower rate. However, if this was the case and CR aging was simply delayed AL aging, then we could easily predict that every age-related change seen during AL aging would occur with similar direction and magnitude and at proportionately the same time relative to total lifespan in CR aging. Most of these predictions could be imagined to hold for some species undergoing idealized temperature-related aging but do not apply for diet-related aging. Instead, several changes over time are clearly diet-specific.

It is important to note that extension of lifespan is positively related to a decreasing calorie intake along a continuum down to a lower calorie limit. Thus under intermediate calorie intakes, intermediate senemorphs are to be expected. While this remains to be confirmed, here we simplify the discussion and consider, as is usually the case with most CR studies, solely the diet-related adaptations at the two extremes: we consider AL aging as the “normal standard” for the fastest aging senemorph because it is adapted to optimize reproduction (analogous to the reproduction-triggered accelerated death in semelparous species); we consider optimized CR aging as the “normal standard” for the slowest aging senemorph because optimized CR aging maximizes lifespan.

Examples accounting for these distinct senemorphoses include:

1. Oxidative damage. The positive relationship between aging and the accumulation of oxidative damage is well known (Finkel and Holbrook 2000). However, CR is able to suppress completely the age-related accumulation of oxidative damage in some tissues (Lass et al. 1998) (Fig. 4a). More remarkably, switching old AL mice to a CR diet can reverse the age-related accumulation of oxidative damage in some tissues (Forster et al. 2000) (Fig. 4a). If CR was simply delayed AL aging, a slowed rate of accumulation of oxidative damage in all tissues rather than a maintenance or a reversal effect would be expected.

**Fig. 4:**
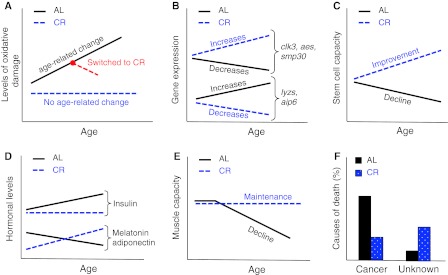
CR and AL are different aging processes which are characterized by distinct senemorphisms. **a** Accumulation of oxidative damage during CR and AL aging: in some cases no age-related change is observed during CR aging (Lass et al. 1998) (*blue line*). In other cases, switching old AL animals to CR (*red line*) may even reverse the accumulated damage (Forster et al. 2000). **b** Depending on tissue (in this case liver) some genes like *clk3*, *aes* and *smp30* increase their expression during CR aging, but decrease their expression during AL aging. The opposite also can occur for example with *lyzs* and *aip6* genes (Son et al. 2006). **c** The repopulation capacity of stem cells is well known to decrease during AL aging. Interestingly, this capacity improves during CR aging (Chen et al. 2003). **d** Most hormones levels remain different in AL versus CR aging. For example insulin levels increases during AL aging, but show no age-related change during CR aging. Adiponectin seems to increase during CR aging, but decrease during AL aging and the same may be true for melatonin (Chiba et al. 2002; Dhahbi et al. 2004; Heilbronn and Ravussin 2003; Roth et al. 2001; Walford et al. 2002). **e** Fitness loss during CR aging is very different to fitness loss during AL aging. CR animals seem to keep their muscle function until the end of life, while AL animals gradually lose muscle function (Hepple et al. 2005). **f** AL and CR animals die for different reasons; most AL mice die due to cancer, while higher frequency of CR animals die due to unknown causes (Shimokawa et al. 1993)
2. Several genes that show age-related increases or decreases in expression during AL aging, show a different or even an antagonistic expression pattern during CR aging (Cao et al. 2001). In some cases the divergence between expression patterns increases as the period of CR lengthens (Fig. 4b). Interestingly, most of the genes that show a difference in expression are related to processes implicated in longevity (e.g. oxidative stress, inflammation, DNA repair, chaperones, metabolism, apoptosis, cell senescence, protein turn-over) (Cao et al. 2001; Coyle and Kroll 2008; Labinskyy et al. 2009; Swindell 2009). These age-related differences in gene expression profiles alone strongly support the existence of diet-related senemorphism.
3. Stem cells lose their repopulation capacity during AL aging. Unexpectedly, short-term CR increases this potential; moreover long-term CR increases it further (Chen et al. 2003) (Fig. 4c).
4. As most hormones are involved in growth, homeostasis and lifespan potential, it is not surprising that they are strongly affected by CR. The hormonal differences between animals undergoing AL and CR are well known. A number of studies have shown hormone levels that move in opposite directions over time in AL versus CR. For example, during AL aging, levels of hormones such as leptin increase whereas adiponectin and melatonin decrease, but possibly are driven in the opposite direction during long-term CR aging (Bedard et al. 2010; Chiba et al. 2002; Longo and Fontana 2010; Roth et al. 2001) (Fig. 4d). Hormones such as insulin, IGF-1, DHEA, GH, ghrelin and cortisol are kept at healthy levels in CR animals (Heilbronn and Ravussin 2003) and shifts can occur in some of them when diet is switched from AL to CR (Dhahbi et al. 2004; Walford et al. 2002). Therefore most hormonal changes (or lack of changes) during senescence are specific to the period of diet and not purely due to chronological age or current diet status. Similarly, reproduction itself triggers the stress-related hormonal changes of accelerated deterioration that lead to semelparous death (Humphries and Stevens 2001; Oakwood et al. 2001; Stein-Behrens and Sapolsky 1992; Wingfield and Sapolsky 2003).
5. The rate of fitness loss (deterioration in skin and hair condition, arthritis, cataracts and other sensory deterioration, muscle weakness, mobility, locomotion, cage climbing, sociality, etc.) in AL animals also follows a different pattern, varying in extent and relative time of onset when compared to CR animals. Therefore, there is no reason to expect to be able to predict the age of degeneration of a particular trait in CR through comparison of its degeneration under AL (e.g. although old AL mice show deterioration in muscle power, even very old CR mice do not). This uncertainty automatically invalidates the assumption that CR aging is a delay of AL aging. (Dubey et al. 1996; Fowler et al. 2002; Hepple et al. 2005; Someya et al. 2007) (Fig. 4e).
6. Cause of death: As seen for breeding versus non-breeding semelparous animals and workers versus queens of social insects, AL and CR animals die from different causes (Shimokawa et al. 1993) (Fig. 4f). This is to be expected only if the two processes are fundamentally different rather than differing only in the speed at which they occur. Where causes of death for CR animals are known, their frequency of occurrence does not follow the same pattern as in AL animals (Sell et al. 2000). Thus, CR is not a delay of AL; the genetic component of the CR program is fundamentally different, leading to a different aging process over time (senemorphism). The outcomes of these aging processes are two biologically different organisms (Fig. 4). In comparison to their respective younger ages, older AL and CR animals have undergone a clearly different senemorphosis, which is only possible due to their longevity plasticity encoded by the genome. From the nature of the genes affected, CR, would be expected to have an effect analogous to that observed in queens of eusocial insects and captive semelparous animals, namely a life-prolonging effect, while AL would have an antagonistic effect akin to that seen in social insect workers and breeding semelparous animals, namely shorter lifespans (Figs. 1, 2, 3). This similarity may not be a coincidence: It may indicate that, as in caste-related and reproduction-related senemorphism both the longer and shorter lifespans of these diet-related senemorphic strategies are specific adaptations to the niche. Below we highlight the importance of the concept of senemorphism for biological research.

## Implications: distinct senemorphisms—distinct approaches

Here we consider the implications of diet-related senemorphism on perceptions of aging and research in the field.

Although CR research has progressed considerably in the past 100 years, the intuitive idea that CR is a delay of AL is still common. This has inhibited our ability to understand both aging processes. It is clear that the amount of calories consumed affects the gene expression profile, which distinctly modulates senemorphosis in most species (species that respond to CR). In other words, multiple senescence patterns modulated by the genome in response to food intake have evolved. This idea has a profound and important effect on how we think, study and analyze aging and age-related diseases. It is most likely the case that the longevity plasticity seen in senemorphic strategies is an adaptation to different environmental conditions; similar ideas have been described (Demetrius 2005; Carnes 2011). Thus, fully fed (AL) animals cannot simply be considered as a standard control for aging studies, an idea previously discussed by Le Bourg (2003). Note that, as elegantly described by Demetrius (2005), different species most likely had adapted divergent mechanisms to increase lifespan under CR conditions. Consequently, it is probably the case that different species also manifest distinct side effects of optimized reproduction under AL conditions. In agreement with Demetrius (2005), we support the idea that the evolution of senemorphic pathways in different species is most likely related to the species-specific life history. Therefore, if diet-related longevity is associated with species-specific metabolic pathways rather than the metabolic rate itself, comparisons between species should be carefully conducted.

Since the amount of food intake alters the metabolism, physiology and the aging process itself, generating different phenotypes, it is crucial for biological studies mainly associated with evolution, aging and age-related diseases to take food into account. As a minimum, the following should be considered: (1) quantity of food should always be measured; (2) a pair feed control group should always be conducted; (3) the same experiment should be carried out under at least the two extreme calorie intakes conditions. For example, the effectiveness of a drug or treatment could depend on high-level expression of SMP30 or stem cell potential. Thus the effectiveness would be enhanced in animals subjected to long-term CR as they show higher levels of SMP30 and stem cell potential compared to young individuals (Fig. 4b, c). Conversely it may be ineffective for animals subject to long-term AL as their levels decrease below those of the young individuals (Fig. 4b, c). In summary, what is true for individuals/populations under long-term (“normal”) AL may be completely false for individuals/populations under long-term CR.

Each diet-related senemorph (even if it is one “snapshot” from a continuum of phenotypes) has its own age-related biology and will most likely have its own fitness value from an evolutionary perspective. Therefore we suggest that all biological studies should take into account which diet-related senemorph is being studied. For instance, by analyzing ant workers we cannot expect to understand ant queens: each morph has a different biology and is adapted to different environments.

**Fig. 5:**
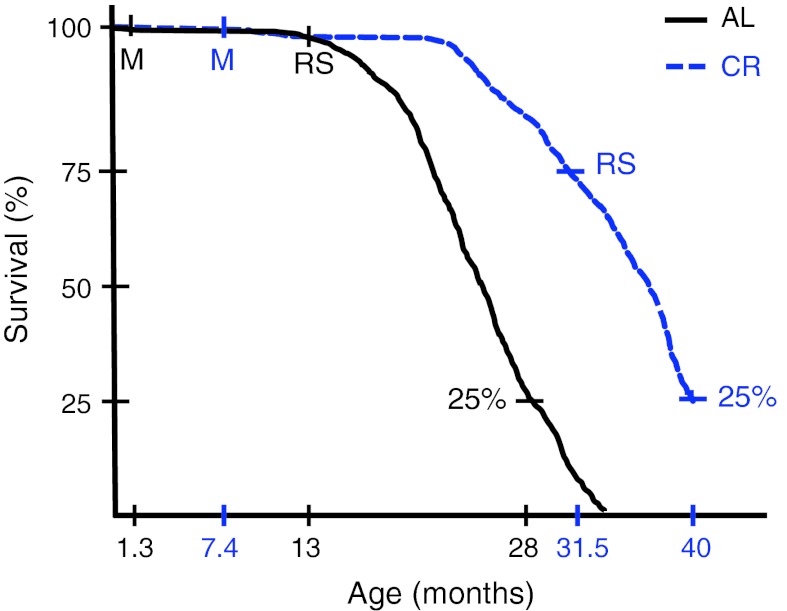
Timing of developmental stages differs in AL and CR rats (adapted from Merry and Holehan 1979). CR does not merely delay the aging process itself: Compared to the AL group, CR lengthened the maturation (*M*) period by 470 %, lengthened the period from maturation to RS by 100 % and reduced the period from RS to 25 % survival by 50 %. Although these animals are from the same species, CR has apparently acted on energy sensing mechanisms to modify several important genetic pathways related to reproduction and longevity. These AL and CR animals differ more physiologically and metabolically than closely related species which are aging while sharing a common diet. Thus comparison of animals with the same chronological age but on different diets may not be the correct comparison to make. This is because the changes related with life-prolonging effects of CR will be masked among the changes related to delayed growth and senescence

CR and AL aging generate different age-related phenotypes, therefore the way in which CR aging is investigated in comparison to AL becomes of vital importance. CR animals for example, should be studied until their natural death and not only for the duration of a “normal” (AL) lifespan. Data from old CR animals should always be compared with young CR animals and not with young AL “control” animals [interesting examples of experimental designs can be found in the literature (Cao et al. 2001; Fontana et al. 2010; Hepple et al. 2005; Lass et al. 1998)]. When AL and CR animals are compared, it would be preferable to compare animals at the same developmental stage rather than the same chronological age (as is done when carrying out inter-species comparisons; Fig. 5). If we compare animals with the same chronological age, the life-prolonging effects of CR will be confounded with the changes related to delayed growth and senescence. For example, comparing a CR animal at 7.4 months old (just matured) with an AL animal at 1.3 months old (just matured) would easily distinguish between the changes related to delayed maturation and the changes related to the life-prolonging affects of CR; such as, increases of stem cell potential or SMP30 (avoiding wrong conclusions) (Fig. 4). The developmental stage itself may dictate the optimal level of CR; there is no reason to feed the CR group according to the AL chronological requirements, if the duration of growth, maturation and reproductive senescence (RS) varies asymmetrically between the two groups (Fig. 5). The calorie intake being dynamically related to AL developmental stage rather than AL chronological age may determine the real maximal lifespan and has not been investigated to date. This approach also would allow facile identification of age-related changes commonly shared by CR and AL animals and, importantly, distinguish the life-prolonging effects of those changes specifically related to the rate of aging from those related to senemorphism.

Because CR aging causes a cascade of specific age-related changes over time it would be reasonable to search for specific diet-related biomarkers of aging (Fig. 4). For instance, age-related decreases in stem cell potential within the AL group seems be an anti-cancer strategy. Consequently, it could be the case that age-related decreases of stem cell potential within the AL group are correlated with longer lifespan. CR animals increase the expression of anti-cancer genes, allowing increases of stem cell potential to enhance regeneration without promoting cancer. In this case, in contrast to the AL group, increases of stem cell potential within the CR group could be correlated with better regeneration and longer lifespans. We strongly support the idea that biomedical research will continually have difficulties to find a reasonable aging biomarker for humans while data from individuals with completely different calorie intakes are not segregated.

It is likely that CR will provide similar benefits to humans as seen in other mammals, however, following a CR regimen is difficult in areas where food is plentiful. Therefore a CR mimetic drug is an attractive option. Putting aside the fact that the changes seen in CR are multifaceted and may not be easy to emulate with a single therapeutic, diet-related senemorphisms raise another serious hurdle: Because AL and CR aging are different processes with widely differing biochemistries, a drug which mimics one part of the CR pathway but in an AL background may have unpredictable effects. For example: the age-related increase of stem cell potential in CR animals could be responsible for the optimized regeneration in these animals. Would it be sensible to mimic increases of stem cell potential in AL animals? Possibly not; CR increases stem cell potential but also increases protection against cancer. Under AL, however, protection against cancer decreases. A CR mimetic drug that increases stem cell potential in AL animals could quite possibly be cancer-inducing.

## Final considerations

We cannot regard aging as a single process, rather the aging phenotype depends on how environmental conditions influence gene expression that will modulate a specific senemorphosis. This has important experimental implications. For example in aging mice we can ask questions such as: do most aging mice develop cancer? Do stem cells lose their efficiency? Does the level of senescent marker protein 30 decrease? Do muscles weaken? The answers are “yes” in all cases for the fully fed (AL) mice phenotype and “no” in all cases for the optimized CR mice phenotype (Fig. 4). These distinct senemorphs are not related to the current dietary status, but to the long-term accumulation of changes under different dietary conditions. Noticeably, most age-related changes in the CR phenotype remain disproportionate to or run in an opposite direction to changes seen in the AL phenotype (Fig. 4). Much more experimental work needs to be carried out to further understand the differences between the two longevity strategies.

Previously published experiments on aging and evolution may also benefit from reanalysis in the light of life-history senemorphism. We expect that an awareness of the existence of diet-related senemorphic processes will accelerate biomedical research and specifically help us to understand the causes of natural death and death by disease. We believe the characterization of distinct age-related biology (senemorphism) as an adaptation to specific diets creates a new perspective on philosophy, evolution and biology.
